# Supplementary material for: An elastic-plastic solution for the optimal thickness of a frozen soil wall considering an interaction with the surrounding rock
Source: PLoS One. 2022 Apr 26;17(4):e0267014. doi: 10.1371/journal.pone.0267014 (PMC9041808; doi:10.1371/journal.pone.0267014)
Supplement: Attachment — Submitted filename: Response to Reviewers (PONE-D-22-04940_R1).docx [file pone.0267014.s001.docx]

**Responses to Reviewers**

**Manuscript Number:** PONE-D-22-04940_R1

**Manuscript Title:** An Elastic-plastic Solution for Optimal Thickness of Frozen Soil Wall Considering Interaction with Surrounding Rock

First of all, we would like to express our sincere thanks to the reviewers for their constructive comments and thoughtful suggestions. The following is a point-to-point response to the reviewers’ comments. Note that the corresponding line numbers in the revised manuscript with track changes have been updated and marked by colored font according to the reviewers’ advice. The reds are corrections by the authors, and the greens are from the scientific editing by AJE.

**Reviewer #1:**

**[Comment 1-1]:**

Establish a specific research goal.

**[Response 1-1]:**

The abstract and the section of Introduction have been modified according to the reviewers’ suggestions, to improve the expressions, with noting the research purpose of this study: “to provide the optimal thickness of the frozen soil wall for excavation using technology of artificial ground freezing” and “a rigorous elastic-plastic solution of cavity contraction is proposed using a non-associated Mohr-Coulomb failure criterion, …, with considerations of the interaction between frozen soil wall and the surrounding soil/rock”.

**[Comment 1-2]:**

Clearly define the type of soil used in the analysis using one of the commonly used classification systems.

**[Response 1-2]:**

It is noted that the proposed solution is applicable to all soil types, as long as the behaviour of soil or frozen soil can be captured by Mohr-Coulomb criterion. This comment has been added to the end of Section3, with “It is noted that the proposed solution is applicable to all soil types, and the mechanical responses of the unfrozen and frozen materials are characterized by the Mohr-Coulomb failure criterion.”.

As to the case study in Section 4, the sinking shaft in Tertiary clay is adopted for analysis. According to the Unified Soil Classification System, the original soil belongs to CL for clay soil with low plasticity. Related information has also been provided in the text, with “According to the Unified Soil Classification System, the soil can be termed as clay soil with low plasticity (CL).”.

**[Comment 1-3]:**

What is the numerical analysis technique or program?

**[Response 1-3]:**

The proposed solution was formulated based on Matlab for calculation, while the numerical simulation using Abaqus was also conducted to validated the analytical solution , as shown in Fig. 3. Some relevant information have been provided as: “A Matlab-based code is then programmed to calculate the distributions of stresses and strains after cavity contraction, and to provide the optimal thickness of the frozen soil wall for excavation.” and “Numerical simulation using Abaqus-based finite element model with the calculated optimal thickness of FSW was conducted for validation of the proposed analytical solution”.

**[Comment 1-4]:**

The cohesion of the soil used in the analysis has a very high value.

**[Response 1-4]:**

The cohesion value (3.5 MPa) in this paper is for the frozen soil at -20 ℃, based on data of experimental tests from the literature (Yang et al, 2013), rather than typical unfrozen soil. That’s why the value is relatively high.

Based on data from the following references, this value can be considered as normal.

1. Paper ‘Experimental Study of Frozen Soil Mechanical Properties for Seismic Design of Pile Foundations’ from ‘Cold Regions Engineering 2012’: ultimate strength of frozen soil is about 3.2 MPa.
2. Paper ‘Mechanical properties of seasonally frozen and permafrost soils at high strain rate’ from ‘Cold Regions Science and Technology’: ultimate strength of frozen soil (T<10 ℃) is between 2.3~7.1 MPa.

Relevant comments have been provided in the text to explain the high value of cohesion.

**[Comment 1-5]:**

The modulus of elasticity of the soil used in the analysis is exceptionally high.

**[Response 1-5]:**

Again, the properties of frozen and unfrozen soils are based on data of experimental tests from the literature (Yang et al, 2013). It is noted that the elastic modulus of unfrozen soil was noted as ‘300 MPa’ by mistake. It should be corrected to ‘100 MPa’, which was used in the following calculation. Compared to the typical value of Young’s modulus (30~60 MPa) for clays with low plasticity (CL), it is high. However, the soil in the case study is embedded in 500 m depth, and the large confining stress results in high Young’s modulus for compacted soils. It is therefore reasonable to get 100 MPa of elastic modulus for unfrozen soil. Some references also show larger ranges:

The elastic modulus of frozen soil is ‘300 MPa’, which is reasonable for soil at -20 ℃. By checking some relevant literature, we can easily find similar value of elastic modulus. For example, Paper ‘Experimental Study of Frozen Soil Mechanical Properties for Seismic Design of Pile Foundations’ from ‘Cold Regions Engineering 2012’ reported the Young’s modulus of frozen soil varies between 270 MPa and 320 MPa.

[https://ascelibrary.org/doi/epdf/10.1061/9780784412473.047]

Relevant comments have been provided in the text to explain the high value of modulus.

**[Comment 1-6]:**

E1 = 300 mPa for FSW in line 222, and E2 = 300 mPa for surrounding in line 223. How?

**[Response 1-6]:**

We feel sorry for this typing mistake. The elastic modulus of frozen soil is 300 MPa, while the magnitude for unfrozen soil is 100 MPa. These values have been explained in the last question, and E2 value has been corrected in the revised paper.

**[Comment 1-7]:**

Line 271, "C1 variation between 0.35 and 0.35 MPa." How?

**[Response 1-7]:**

Thanks for pointing out this mistake. It was also a typo-mistake, and the correct range should be between 0.35 and 35 MPa, as revised in the paper.

**[Comment 1-8]:**

Extend the discussion by mentioning the numerical results in conclusion.

**[Response 1-8]:**

Thanks for your suggestion. The conclusion has been revised with additional discussion on numerical simulation: “Numerical simulation based on Finite Element Method was conducted to validate the solution regarding to the distributions of stress, strains and displacements. The developed solution was then confirmed by the good comparisons with results of numerical simulation.”.

**[Comment 1-9]:**

paraphrasing a few sentences as shown in the attached file

**[Response 1-9]:**

Although I have not received any attached file from the Journal, we have revised the technical writing of this paper thoroughly. Hope the revised paper with the current version has met all requirements for publication.

**Reviewer #2:**

**[Comment 2-1]:**

The abstract should state the major points of your research briefly and explain why your work is important, your purpose, how you went about your project, what you learned, and what you concluded. An abstract is often presented separately from the article, so it must be able to stand alone.

**[Response 2-1]:**

The abstract of this paper has been modified substantially, according to the constructive suggestion.

**[Comment 2-2]:**

The introduction serves the purpose of leading the reader from a general subject area to a particular field of research. It establishes the context of the research being conducted by summarizing current understanding and background information about the topic, stating the purpose of the work in the form of the hypothesis, question, or research problem, briefly explaining your rationale, methodological approach, highlighting the potential outcomes your study can reveal, and describing the remaining structure of the paper.

**[Response 2-2]:**

Thanks for your constructive suggestion. The section of Introduction has been modified accordingly, especially to strengthen the statements about the background, limitations and the purposes of this study. Also, the novelty of this study has been introduced and the general structure of this paper has been briefly described.

**[Comment 2-3]:**

Remove any the figures from the introduction.

**[Response 2-3]:**

The original Fig. 1 in the section of Introduction has been removed.

**[Comment 2-4]:**

A flowchart should be added to the article to show the research methodology.

**[Response 2-4]:**

A flowchart of the analytical solution has been added for calculation of optimal thickness of FSW.

**[Comment 2-5]:**

The necessity and innovation of the article should be presented to the introduction.

**[Response 2-5]:**

Thanks for pointing out this flaw of the manuscript. In the revised Introduction, the drawbacks of the existing solutions have been further explained to highlight the necessity of this study. It is now noted as: “However, the current solutions neglect the effects of friction induced dilation, which is typically significant to the geomaterials. Large deformation caused by excavation is usually assumed as infinitely small, that could result in large errors in the estimation of optimal thickness of the FSW. The outer confining stress of the FSW is a changing parameter during construction, and plays a vital role in the FSW-soil interaction system. It is therefore necessary to improve the analytical method with considerations of both cohesive-frictional behaviour of frozen soil and interactions between the FSW and the surrounding soil.”.

The innovation of this study has also been strengthened in the last paragraph of Introduction. Some additional comments are: “This paper attempts to provide a rigorous elastic-plastic solution of cavity contraction for the design of optimal thickness of the frozen soil wall with consideration of interaction with the surrounding soil.”; “For soil in the plastic region, large strain analysis is incorporated within the solution to account for the excavation induced large deformation, while the assumption of no volumetric strain is eliminated.”; “The innovation of this study is also instantiated by the proposed optimal thickness of the frozen soil wall”.

**[Comment 2-6]:**

The paper language and punctuation need revision by an expert.

**[Response 2-6]:**

The language of this paper has been revised by a native speaking expert. Additionally, the suggested professional scientific editing service helps to improve the writing of this paper, with green corrections. Hope it is better now to be accepted for publication.

**[Comment 2-7]:**

Sections 2 Problem definition and 3 Analytical solution of cavity contraction, should be subsection under the heading "Methodology".

**[Response 2-7]:**

The structure of this paper has been modified according to the suggestion. Section of “Methodology” has been added to merge ‘Problem definition’ and ‘Analytical solution’.

**[Comment 2-8]:**

Enhancing the study with realistic images of experimental work.

**[Response 2-8]:**

As this study focuses on the analytical study, and no experimental work was conducted specifically for this study. All parameters were selected based on previous studies from literature. Comments have been provided to explain their reasonability.

However, a case study was used to set the parameters in Section 3, and the background of this case study has been further introduced, with typical experimental results of a sample from this site. Hope this additional figure could assist the understanding on the basic mechanical response of frozen soil at high stress condition.

**[Comment 2-9]:**

Consider enhancing the used references as they are few and old.

**[Response 2-9]:**

The references of this paper have been enhanced as suggested. Some recent studies related to AGF and frozen soils have been included.

**[Comment 2-10]:**

Add the DOI to all references.

**[Response 2-10]:**

Thanks for your suggestion. DOI for all references have been added, if available. DOI for some papers are not available, and ISBN numbers are given instead for textbooks.
